# Use of Polylactic Acid Dermal Matrix for the Management of Wounds with Exposed Avascular Structures

**DOI:** 10.3390/jcm15010003

**Published:** 2025-12-19

**Authors:** Mario Aurelio Martínez-Jiménez, Ana Lorena Novoa-Moreno, Victor Manuel Loza-González, Rafael Pérez-Medina-Carballo, Patricia Aurea Cervantes-Báez

**Affiliations:** 1 Burn Unit, Regional High-Specialty Hospital “Dr. Ignacio Morones Prieto”, San Luis Potosí 78290, Mexicopaty.cervantesb@hotmail.com (P.A.C.-B.); 2 Institutional Doctorate in Engineering and Science of Materials, Autonomous University of San Luis Potosí, San Luis Potosí 78290, Mexico; 3 Integrated Program in Neuroscience, Faculty of Science, McGill University, Montreal, QC H3A 0G4, Canada; rafael.perezmedinacarballo@mail.mcgill.ca

**Keywords:** avascular, wounds, polylactic acid, synthetic biomaterials, tissue regeneration

## Abstract

**Background:** Wounds with an avascular component represent a significant challenge in medical care due to impaired blood flow. Synthetic matrices, such as poly-lactic acid (PLA), have demonstrated promising results in promoting wound healing in complex wounds, including those with restricted blood supply, such as diabetic foot and venous leg ulcers. **Objective:** This case series presents the outcomes of five patients with wounds containing exposure of avascular components, of various etiologies successfully treated with PLA matrices. **Case description:** Five patients presented complex wounds involving exposure of bone, tendon, fascia, or osteosynthetic material. Wound bed preparation included debridement followed by PLA application covered with additional layers (non-adherent dressing, absorbent dressing, and compression bandage) as needed. Weekly assessments were conducted until full wound closure was achieved. **Results:** All cases showed successful outcomes, with PLA promoting granulation tissue formation and re-epithelialization, contributing to wound closure. One patient required skin grafts for complete healing. No local infections were reported before or after PLA application. **Conclusions:** PLA matrices are a practical and effective option for managing complex wounds, promoting tissue regeneration and optimizing wound bed quality for skin grafts or flaps. While these findings are promising, further studies are needed to confirm the broader applicability and efficacy of PLA in the management of wounds containing exposure of avascular structures.

## 1. Introduction

Wounds with avascular components, such as exposed bone, joint surfaces, tendons, and orthopedic hardware, pose significant challenges in wound care due to the lack of blood flow to these structures. This absence hinders revascularization and the formation of regenerative tissue, which is crucial for advancing smoothly along the stages of healing. Advancing from a starting stage of inflammation to a proliferation stage where granulation tissue starts to develop, the neoformation of blood vessels is determining [1].

Historically, the gold standard for treating such complex soft tissue defects has been the transfer of vascularized tissue through microsurgical procedures or regional muscle, skin, or fascia transposition [2]. However, recent technologies such as negative wound pressure therapy and cellular, acellular and matrix-like products (CAMPs) have allowed the closure of these defects through less invasive procedures via a combination of wound bed temporization and skin grafting [3,4]. A systematic review performed by Iorio et al. analyzed 13 primary studies with a total of 432 patients and concluded the use of CAMP’s in chronic and acute injuries where there is exposed bone, tendon, and/or muscle, is a viable and effective option for tissue coverage [5].

CAMPs are a heterogeneous group of advanced wound care therapies comprising living cells, tissues, and/or engineered materials derived from diverse sources. These products are designed to promote wound healing and tissue regeneration by providing bioactive components, growth factors, and structural support to wound healing [6]. Within CAMPs, synthetic dermal closure matrices, a novel category of alloplastic skin substitutes, are increasingly used for wound closure. Specifically, poly-lactic acid (PLA) wound closure matrices have consistently demonstrated favorable closure outcomes for patients with complex wounds and burns by restoring the interaction of the key elements involved in the wound-healing process [7,8]. Most of these outcomes can be directly attributed to the fact that the degradation product of PLA matrices is lactate, which, in turn, is a potent signaling molecule that enhances neo-angiogenesis in wounds [9].

Given these promising results in wounds from etiologies like diabetic foot ulcers that share the characteristic of having impaired vascular supply, we believe PLA matrices have significant potential for treating wounds with avascular components [10,11,12] like the ones shown in this paper. Here, we present a case series of five patients successfully treated with a novel PLA dermal matrix to enhance wound repair over complex soft tissue defects with exposed avascular structures.

## 2. Case Description

Five patients (three females, two males) with wounds of various etiologies and exposed avascular structures were treated with PLA matrices at the Wound Clinic of a tertiary level trauma care center in the city of San Luis Potosi, Mexico. The management protocol for these wounds included a comprehensive clinical and wound assessment, with particular attention to the use of medications affecting wound healing, nutritional optimization when needed, and assessment of the vascular and infectious status of the wounds following the “prepare to repair” paradigm [10]. These cases, represent all individuals seen during the study period who met the predefined eligibility criteria; presence of exposed avascular structures such as bone, tendon, or ostheosynthetic material; absence of major vascular pathology, ruled out using a combination of infrared thermography, ankle-brachial index, and clinical evaluation [13] and bacterial contamination or infection excluded through fluorescence-based assessments and bacterial culture [11].

**Figure 1:**
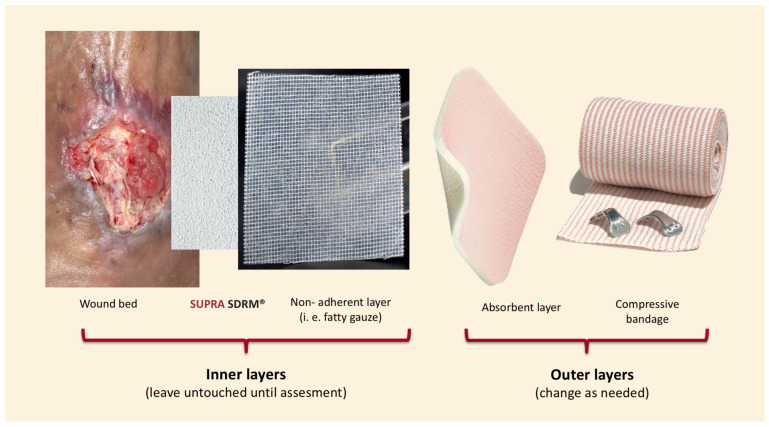
“Sandwich technique.” Description of the method used for covering a wound by layering multiple materials.

**Figure 2:**
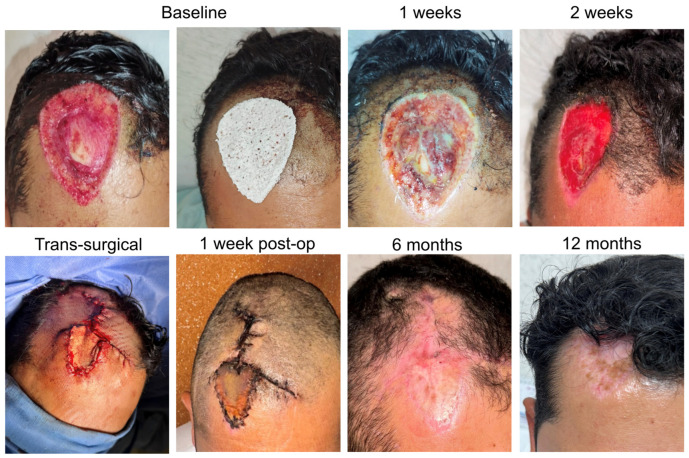
Sequential clinical evolution of Case 5 showing progressive granulation over the exposed structure after PLA matrix application, residual matrix during biodegradation, and final defect coverage with split-thickness skin graft. Complete wound closure and skin maturation were achieved, with no complications observed during the 6- and 12-month follow-up.

Wound bed preparation included surgical debridement and hemostasis, followed by the application of PLA matrices (Supra SDRM, PolyMedics Innovations, Kirchheim unter Teck, Germany) using the “sandwich technique” (Figure 1), this standardized method ensured consistency across cases and minimized variability. Briefly, the PLA matrix was applied directly onto the wound bed without extending beyond the edges. For undermined areas or deep soft tissue defects, the material was layered in up to two or three layers depending on the depth of the wound; however, this multilayer was only used in Case 5 (Figure 2). Next, a self-adherent, non-contact dressing (Supra Net, Polymedics Innovations) was added, and an outer layer of absorbent dressings (i.e., gauze pads or silver foam dressings) was applied on top. Finally, a mild compression bandage was used to secure all the layers below. This dressing technique allows the changing of the outermost layers as often as needed without disturbing the inner layers containing the PLA matrix and non-contact layer. Outer dressing changes were performed every seven days, and wound assessments were performed on a weekly basis until healing. In all cases, PLA matrices were applied only once and remained untouched until complete degradation, which occurred between days 7 and 14, depending on the moisture of the wound and the number of layers applied. All patients achieved complete defect closure, either through secondary intention or following additional surgical intervention. Patients were followed for a minimum of 6 months after wound closure, with extended follow-up up to 12 months in selected cases. Patients were discharged once full skin maturation was achieved. No recurrences or late complications were observed during the follow-up period.

### 2.1. Case 1

**Figure 3:**
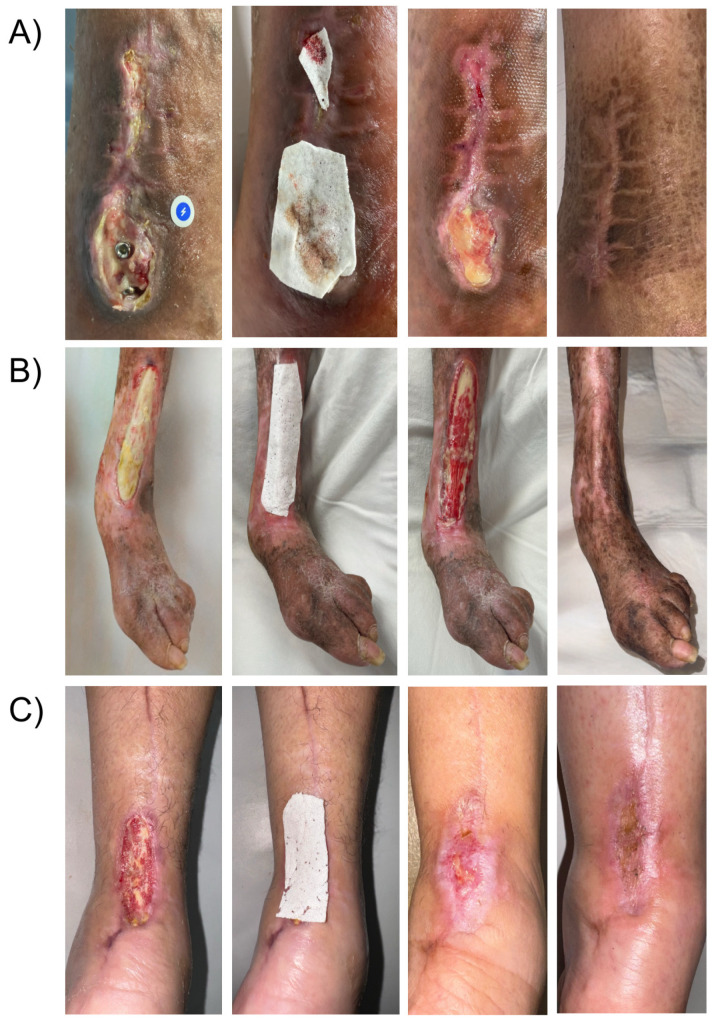
Sequential presentation of lower-leg injuries: (**A**) tibial fracture wound with osteosynthesis material, (**B**) friction wound with exposed tendon, and (**C**) postoperative wound showing Achilles tendon exposure.

A 62-year-old male with no significant medical history developed a surgical site infection and dehiscence on day 5 after fixation of an exposed tibial fracture (Figure 3A). The wound bed was necrotic and covered with slough. After undergoing drainage, debridement, and a one-week course of IV antibiotics, exposed osteosynthetic material was identified and was referred to our service 3 weeks after the surgical procedure was done. Negative culture results were obtained before applying PLA matrices to the wound bed covering the fixation hardware. By week five, granulation tissue covered the metal implants, leading to complete healing of the wound at week eight with no major scarring or mobility limitation after a 10-month follow-up.

### 2.2. Case 2

An 82-year-old female patient with a mild malnutrition status (BMI < 20), bedridden due to a previous stroke, and exhibiting signs of dementia, presented with a one-week-old wound on the anterior region of the left leg exposing the extensor hallucis longus tendon caused by rubbing one leg against the other (Figure 3B). During the initial assessment, debridement of the wound and application of a single layer of PLA matrices were performed. Over the following weeks, progressive granulation tissue formation over the peritendon was observed, and by the fourth week, re-epithelialization of the wound edges was completed. The patient was followed for six additional months, during which the healed site had good skin quality and only minor pigmentation changes in the scar tissue.

### 2.3. Case 3

A 56-year-old female with controlled type 2 diabetes mellitus was referred to our clinic for a surgical wound dehiscence following an Achilles tendon repair 3 weeks before (Figure 3C). The wound resulted in significant exposure of the peritendon. PLA matrices were applied to the wound, and outer dressings were changed weekly. Complete wound closure was achieved within 10 weeks, with excellent skin quality and preserved range of motion, suggesting no adherence of the tendon to the skin at the closure site.

### 2.4. Case 4

**Figure 4:**
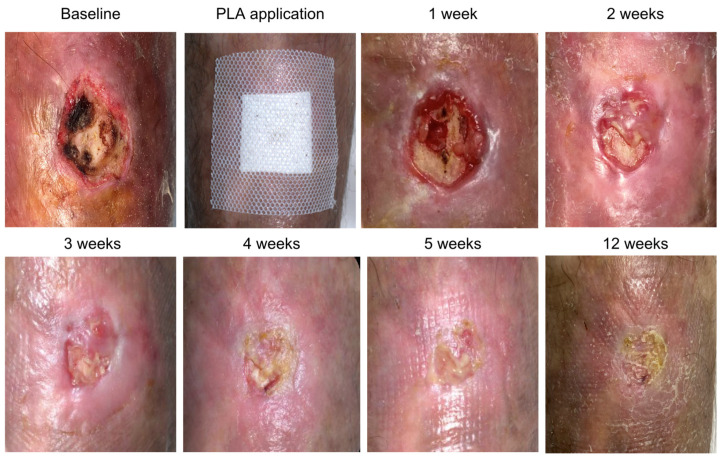
Right leg ulcer with exposed bone.

A 57-year-old female patient with a medical history of uncontrolled diabetes (Hb A1c > 7%) and lupus erythematosus under chronic high-dose corticosteroid therapy, corresponding to >20 mg/day of oral prednisone, was referred to the Wound Clinic after experiencing a fall that resulted in an ulcer on her right leg with exposure of the underlying bone (Figure 4). The ulcer failed to heal with standard wound care management, and the periosteum eventually became necrotic. The patient reported severe pain and expressed concerns regarding potential further tissue damage, ultimately refusing any surgical intervention, such as a tissue flap. The decision to apply PLA matrices was made in response to this, and the material was applied to the wound to cover the defect. Interestingly, over the course of the initial week of treatment, the patient reported pain relief. Over the following course of five weeks, granulation tissue and epithelium formed from the wound edges, leading to complete cover of the bone defect, closure of the ulcer, and successful healing. 

### 2.5. Case 5

A 32-year-old healthy male patient presented to the emergency department with a frontal region trauma caused by a sharp-blunt weapon (machete), resulting in an avulsive injury with exposed bone (Figure 2). The patient was admitted for wound bed preparation, which involved the debridement of devitalized tissue and intraoperative application of PLA matrices. After two weeks of treatment and despite the initial size of the wound, more than 15% of the size reduction was observed without significant tissue contraction. The periosteum was found to be covered with well-vascularized repair tissue, making the patient a candidate for reconstruction using a scalp advancement flap and a full-thickness skin graft over the residual wound site. This large soft tissue defect healed with good skin quality and without adverse sequelae.

## 3. Discussion

The case series presented in this manuscript involves five patients treated with PLA-guided matrices for various wounds with exposed avascular structures. Among these patients, one presented with an avulsive wound in the frontal region that involved exposure of the underlying bone. The remaining four cases involved wounds in the lower extremities, characterized by exposure of osteosynthetic material, bone, tendon, or fascia (Table 1). The cases presented in the current manuscript demonstrate that PLA matrices are suitable material for promoting adequate repair of wounds with exposure of avascular components.

PLA matrices have demonstrated effectiveness in treating other complex wounds, including burns, diabetic foot ulcers, and venous leg ulcers [6]. Yet, evidence regarding their use in soft tissue defects with exposed avascular components remains limited. In recent studies conducted by our group, PLA treatment reduced the time to complete healing by 44% in diabetic foot ulcers and by 95% in venous leg ulcers compared to collagen, and 40% compared to fish grafts. 80% of PLA-treated patients achieved full healing within 12 weeks, as opposed to 33% in the collagen group [12,14]. Other synthetic materials, such as polyglycolic acid and boron-based glass fibrous matrices, have also been reported in case reports addressing the management of this type of wound, but further evidence is needed to establish their efficacy [6]. Polyurethane matrices have also been explored in complex wounds with exposed bone or tendon. A case series by Solanki et al. [15] reported positive outcomes in 25 patients, including 18 with complex wounds, treated with a two-stage reconstruction approach that required skin grafting, although infections were commonly encountered. In our series, one case required skin grafts, but no infections were observed after applying the PLA matrices, suggesting that PLA matrices may offer comparable benefits with potentially fewer complications. In a systematic review performed by Chen HL et al. 14 studies discussing the effects of PLA-based biomaterials in cutaneous wound healing were analyzed concluding PLA-based biomaterials not only promote wound healing but can also prevent infections and reduce pain [16].

The therapeutic effect of the PLA matrices can be mainly attributed to the release of lactate, which acts as a paracrine agent (referred to as a lactormone) with potent signaling effects that promote neo-vascularization through stimulation of vascular endothelial growth factor (VEGF), leading to granulation in the wound bed and facilitating collagen synthesis and fibroblast migration [8,9,15]. Lactate also lowers the local pH levels, which in turn inhibits bacterial growth and prevents matrix degradation caused by bacterial activity [8,15]. Furthermore, the microporous structure of the PLA matrices serves as a supportive scaffold and maintains a moist environment with high vapor permeability [7]. These mechanisms contribute to the preservation of tissue viability and integrity, assisting in secondary closure or preparing the wound bed for definitive surgical repair.

Synthetic matrices, such as PLA, offer a promising, practical, and simple alternative for optimizing the wound bed prior to flap or graft placement, thereby enhancing outcomes, reducing complications, and potentially allowing for spontaneous closure in selected small, non-recurrent wounds.

In this case series, one out of five patients required skin grafts after receiving PLA matrix treatment. Therefore, it is reasonable to propose that PLA matrices could be used initially for one to two weeks to assess wound healing progress, potentially decreasing the need for more complex therapies.

### Limitations

As we present our case series, it is important to acknowledge that our findings may not yet be generalizable and instead, reflect the clinical experiences of practitioners using PLA matrices for wounds with exposure of avascular structures. Even if our results are promising, it remains essential to emphasize the need for further evidence. The absence of a comparator group along with the sample size limits the strength of any causal interpretation. Additionally, because the cases included were those that met specific clinical criteria within out practice, a degree of selection bias cannot be fully excluded. Objective quantitative measurements of wound progression were limited by the retrospective nature of the series and the lack of standardized measurement tools in our clinical setting.

Further research focusing on complex soft tissue defects and comparative studies is necessary to thoroughly evaluate the effectiveness of PLA and support its wider applications in clinical practice.

## 4. Conclusions

This case series highlights the potential of PLA matrices as an effective and practical option for the management of wounds with exposed avascular components. Across five cases involving various complex wound etiologies, PLA matrices promoted granulation tissue formation, supported re-epithelialization, and achieved successful wound closure. The favorable outcomes observed suggest that PLA matrices may represent a promising alternative to more complex therapies, such as negative pressure wound therapy and skin transplantation, while remaining accessible to a wide range of health care providers. While these observations offer valuable insights into the potential applications of PLA for this type of wound, further research is needed to confirm these observations and establish the broader clinical efficacy of this approach.

## Figures and Tables

**Table 1:** Demographic table.

Patient NO	Etiology	Size of the Defect	Characteristics of Defect		Structures Exposed	Time of Evolution	PLA Applications	Frequency of Application	Time to Complete Healing
			Tissue Present	Exudate					
1	Wound defect after tibial fracture repair	13.7 cm^2^	Epithelial 5%, granulation 15%, slough 80%, eschar 0%	Serous, watery, moderate	Ostheosynthetic material	5 days	Once a week	7 times	8 weeks
2	Friction wound in the anterior leg	68.4 cm^2^	Epithelial 0%, granulation 0%, slough 100%, eschar 0%	Serous, watery, moderate	Tendon	7 days	Once a week	3 times	4 weeks
3	Wound defect after Aquiles tendon repair	5.7 cm^2^	Epithelial 5%, granulation 80%, slough 15%, eschar 0%	Serous, clear, minimal	Tendon	21 days	Once week	10 times	12 weeks
4	Traumatic ulcer + LES	3.8 cm^2^	Epithelial 0, granulation 10, slough 10, eschar 40%	Serosanguinous, minimal	Bone	90 days	Once week	3 times	5 weeks
5	Trauma to the head (Machete lesion)	25.3 cm^2^	Epithelial 0%, granulation 80%, slough 0, eschar 0%	Serous, clear, minimal	Bone	1 day	Only once	1 times	2 weeks (skin grafted)

## Data Availability

No new data was created or analyzed in this study. Data sharing is not applicable to this article.

## Abbreviations

The following abbreviations are used in this manuscript:

PLA	Polylactic acid
CAMP’s	Cellular, acellular and matrix-like products
IV	Intra venous
VEGF	Vascular endothelial growth factor
